# Development and validation of a symptom assessment tool for postmicturition dribble: A prospective, multicenter, observational study in Korea

**DOI:** 10.1371/journal.pone.0223734

**Published:** 2019-10-11

**Authors:** Hyun Cheol Jeong, Kyung Tae Ko, Dae Yul Yang, Won Ki Lee, Sang Kon Lee, Sung Tae Cho, Cheol Young Oh, Jin Seon Cho, Jong Keun Kim, Jun Hyun Han, Min Soo Choo, Seong Ho Lee

**Affiliations:** 1 Department of Urology, College of Medicine, Hallym University, Kangdong Sacred Heart Hospital, Seoul, Korea; 2 Department of Urology, College of Medicine, Hallym University, Chuncheon Sacred Heart Hospital, Chuncheon, Korea; 3 Department of Urology, College of Medicine, Hallym University, Kangnam Sacred Heart Hospital, Chuncheon, Korea; 4 Department of Urology, College of Medicine, Hallym University, Hallym University Sacred Heart Hospital, Anyang, Korea; 5 Department of Urology, College of Medicine, Hallym University, Dongtan Sacred Heart Hospital, Hwaseong, Korea; University Medical Center Utrecht, NETHERLANDS

## Abstract

**Objectives:**

Postmicturition dribble (PMD) is a very common symptom in males with lower urinary tract symptoms (LUTS) worldwide, but there is no adequate questionnaire to assess it. Therefore, we developed a questionnaire named the Hallym Post Micturition Dribble Questionnaire (HPMDQ) to assess PMD, and the aim of this study is to validate it.

**Methods:**

A series of consecutive male patients newly diagnosed with LUTS and over 40 years of age who visited any of 5 medical institutions were included. LUTS were assessed in all patients using the International Prostate Symptom Score (IPSS), and PMD was assessed using the HPMDQ.

**Results:**

In total, 2134 male patients aged 40 to 91 years were included in this study. Of these patients, 1088 (51.0%) reported PMD. In the PMD group, the mean values for HPMDQ-Q1, HPMDQ-Q2, HPMDQ-Q3 and HPMDQ total score were 1.39, 1.10, 1.76 and 4.25, respectively. In the non-PMD group, the mean values of these scores were 0, 0.18, 1.52 and 1.58, respectively. The difference in HPMDQ scores between the 2 groups was statistically significant. PMD was significantly associated with the voiding symptoms of LUTS, prostate size and postvoid residual but not with storage symptoms.

**Conclusions:**

The HPMDQ, which consists of 5 questions (frequency, severity, bother, quality of life and response to treatment for PMD), was developed, and its use for assessing PMD is validated in this study. It may be a useful tool for further research and in clinical practice for PMD.

## Introduction

Postmicturition dribble (PMD) is a term used to describe the involuntary loss of urine immediately after an individual finishes passing urine, usually after leaving the toilet in men or after rising from the toilet in women. [1] PMD is classified as a postmicturition symptom according to the standardization of terminology of lower urinary tract symptoms (LUTS) by the International Continence Society (ICS). [1]

Although the exact pathophysiological mechanism of PMD has not been clarified to date, earlier studies suggest that PMD is secondary to a small amount of residual urine in either the bulbar or prostatic urethra that is normally "milked back" into the bladder at the end of micturition. [2,3]

PMD is one of the most prevalent LUTS [4–9] and is known to be one of the most common causes of bother. [4,5,10] However, recent studies on LUTS have focused on the symptoms of voiding or storage, and only a few studies on PMD in the literature are available, despite its high prevalence and potential burden on the quality of life of patients. Furthermore, most studies have focused on the prevalence of PMD using population-based surveys, and there is not much literature on the mechanism in, assessment tool for and treatment of PMD.

To date, many questionnaires, such as the International Prostate Symptom Score (IPSS) and Overactive Bladder Symptom Score (OABSS), have been used to assess LUTS, but there is no clinical tool for the evaluation of PMD. Therefore, we developed a questionnaire for the evaluation of PMD that consists of 5 questions as follows: frequency, severity, bother, quality of life and response to treatment. Furthermore, we validated this questionnaire in this study.

## Materials and methods

### Study participants

This prospective, multicenter, observational study was performed at 5 medical institutions of Hallym Medical Center in South Korea. The study protocol was reviewed and approved by the institutional review board of Dongtan sacred heart hospital (Approval number: DTF 2015–004). This study was performed in accordance with the Declaration of Helsinki and the Ethical Guidelines for Clinical Studies. Data collected during this study were analyzed. All patients provided written informed consent before enrollment. A series of consecutive male patients newly diagnosed with LUTS and over 40 years of age who visited any of the 5 medical institutions between June 2014 and December 2016 were included in the present study. Patients with neurological disorders, urinary tract infections, renal insufficiency, bladder stones, prostate cancer, urethral stricture and previous pelvic surgery, and patients who took medications that were related to LUTS within the previous 4 weeks, were excluded from the study. LUTS were assessed in all patients using a validated questionnaire, i.e., IPSS, which included a quality of life question (IPSS-QoL) [11], and PMD was assessed using the HPMDQ.

### HPMDQ development

To develop a tool for diagnosis and follow-up of PMD at the outpatient clinic, we have undergone a number of specific reviews with several experts who expertise in the area of LUTS. The questionnaires were developed by coordinating the opinions of 12 urologists, each with more than 10 years of clinical experience of LUTS. Using the definition of PMD, we selected four questions (on frequency, severity, bother, and quality of life) and one additional question on treatment response.

The HPMDQ is a self-administered questionnaire that was developed by the authors to evaluate PMD, and it consists of 5 questions concerning frequency (Question 1), severity (Question 1–1), bother (Question 2), quality of life (Question 3) and response to treatment (Question 4) (S1 File). In case of Q4, it is not to be written if it is done before any treatment. We considered the patient to have PMD if the score on question 1 was 1 or more. Each questions had answers on a four-grade response scale (0–3). The HPMDQ total score was defined as the sum of the scores of questions 1, 2, and 3.

### HPMDQ validation study

We calculated Cronbach’s alpha to check the internal consistency and reliability of the questionnaire. We used Cronbach’s alpha > 0.60 as the standard for acceptable instrument reliability.

All patients answered the questions on the HPMDQ and were divided into the PMD and non-PMD patient groups. The primary outcome of this study was differences in the total score and scores for each HPMDQ between the two groups and differences were analyzed by Student's t-test.

For the quantitative analysis, we measured PMD volume by the paper test in only those who agreed to the test. The examiner instructed the patient to place the paper made for the examination inside the underwear before standing. After a few minutes of walking, the amount of PMD that leaked onto the paper was measured. We analyzed the correlation between the paper test and HPMDQ total score by Spearman’s correlation coefficient, r.

The prostate volume was measured by transrectal ultrasound, and the maximum urinary flow rate (Qmax) and postvoid residual were assessed using a uroflowmeter and a bladder scan, respectively. Based on these data, we evaluated the prevalence and bother of PMD and the relationship between PMD and other LUTS/BPH-related factors.

### Statistical analysis

Statistical significance was assessed by Student's t-test, the chi-square test, partial correlations, a multiple linear regression analysis and Spearman’s r using SPSS version 22.0 for Windows (IBM Corp., Armonk, NY, USA). A p-value<0.05 was considered statistically significant.

## Results

### 1. Study participants

In total, 2134 male patients aged 40 to 91 years were included in this study. The mean age of the patients was 62.0±10.9 years. Of these patients, 279 (13.1%) were 40–49 years old, 419 (23.0%) were 50–59 years old, 804 (37.7%) were 60–69 years old and 560 (26.2%) were 70 years old or older. The mean IPSS, IPSS-voiding, IPSS-storage and IPSS-QoL scores were 12.6, 7.2, 5.4 and 2.7, respectively. The baseline characteristics of the study participants are summarized in Table 1.

**Table 1:** Clinical characteristics of patients.

Variable (Mean±SD)	No.of patients	%
Age, years	62.0±10.9	
40–49	279	13.1
50–59	491	23.0
60–69	804	37.7
≥70	560	26.2
Total IPSS	12.6±7.6	
0–7	635	29.7
8–20	1152	54.0
21–35	347	16.3
Voiding IPSS	7.2±5.3	
Storage IPSS	5.4±4.2	
IPSS QoL	2.7±1.6	
Prostate size (ml)	38.9±18.6	
PSA	2.5±4.8	
Qmax (ml/sec)	12.1±8.4	
PVR (ml)	42.1±86.6	

IPSS, International Prostate Symptom Score; PSA, prostate specific antigen; PVR: postvoid residual

### 2. The difference in HPMDQ score between PMD and non-PMD groups

In the PMD group, the mean value of the HPMDQ-Q1, HPMDQ-Q2, HPMDQ-Q3 and total scores were 1.39, 1.10, 1.76 and 4.25, respectively. In the non-PMD group, the mean values of these scores were 0, 0.18, 1.52 and 1.58, respectively. There were statistically significant differences between the two groups for all three questionnaire scores and for the total score (p<0.001, p<0.001, p<0.001 and p<0.001) (Table 2).

**Table 2:** The difference in mean value of HPMDQ score between patients who had PMD or not (A p-value<0.05 was considered statistically significant).

HPMDQ	Q1	Q2	Q3	Total score
PMD	1.39	1.10	1.76	4.25
No PMD	0	0.18	1.52	1.58
p-value	<0.001	<0.001	<0.001	<0.001

### 3. Prevalence of PMD

Of the 2134 patients, 1088 (51.0%) reported PMD. The prevalence of PMD was 51.3%, 52.2%, 55.6% and 41.4% among those aged 40–49 years, 50–59 years, 60–69 years and 70 years or older, respectively. The prevalence of PMD increased with age until the age of seventy, upon which it decreased. (Table 3).

**Table 3:** Postmicturition dribble (PMD) prevalence according to age.

Age	40–49	50–59	60–69	≥70	Total
No.	279	491	804	560	2134
PMD	143	266	447	232	1088
PMD%	51.3	54.2	55.6	41.4	51.0

### 4. Bother of PMD

Of the 1088 patients with PMD, 865 (79.5%) reported bother from PMD. In total, 624 (57.4%), 147 (13.5%) and 94 (8.6%) patients reported minor, moderate and severe bother, respectively.

**Fig 1:**
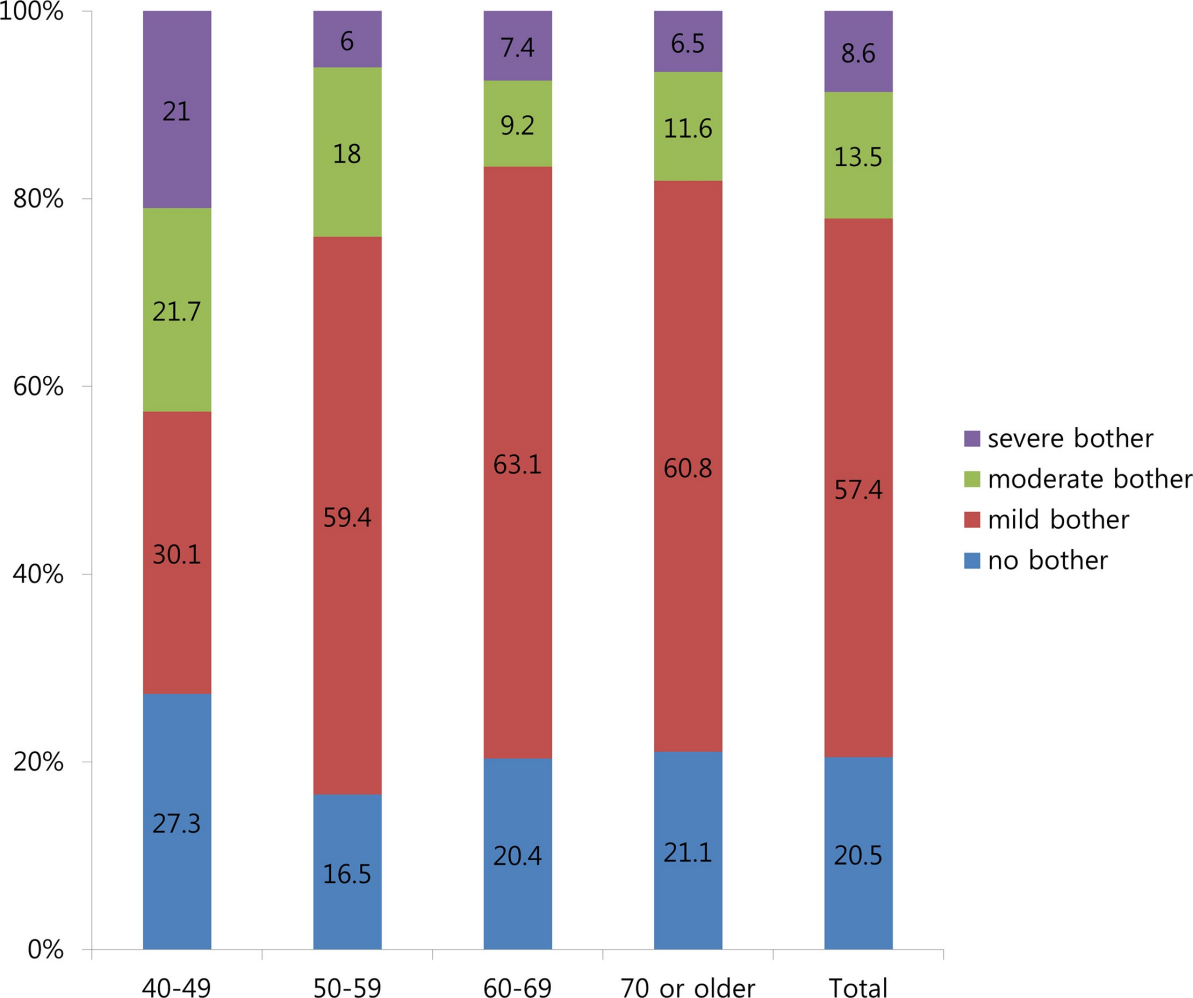
Degree of bother of postmicturition dribble according to age.

The prevalence of patients who reported bother from PMD were 72.7%, 83.5%, 79.6% and 78.9% among those aged 40–49 years, 50–59 years, 60–69 years and 70 years or older, respectively. No significant difference in PMD prevalence was observed among the age groups (chi-square test, p = 0.84). However, in the 40–49 years group, the prevalence of patients who reported moderate or severe bother was significantly higher than that in the older age groups (chi-square test, p<0.001) (Fig 1).

### 5. Association between PMD and paper test

**Fig 2:**
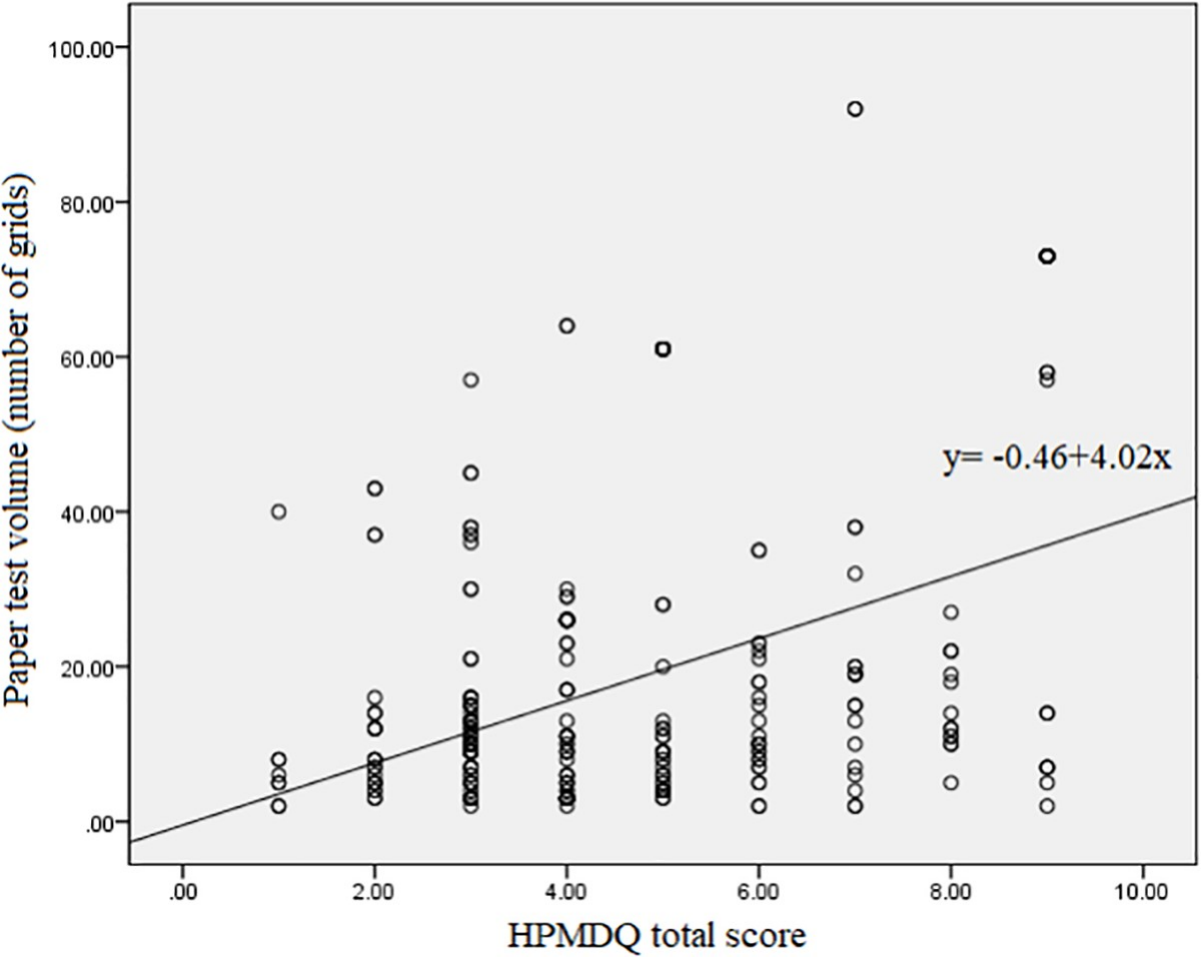
Correlation of paper test volume (number of grids) and HPMDQ total score (y = -0.46+4.02x, y = PMD volume, x = PMD total score). HPMDQ: Hallym PMD Questionnaire.

A total of 371 patients were evaluated and included in the analysis because the paper test was performed in only those who agreed to the test. Correlation analysis showed that all three symptom scores and the total score were significantly positively correlated with the paper test results (y = -0.46+4.02x, y = PMD volume, x = PMD total score) (Fig 2). The results of each question were as follows: PMD 1 (r = 0.38, p <0.001), PMD 2 (r = 0.463, p <0.001), PMD 3 (r = 0.159, p < 0.001) and PMD total (r = 0.417, p<0.001).

### 6. Association between PMD and other LUTS/BPH-related factors

According to the multiple logistic regression analysis, PMD was significantly associated with the voiding symptoms of LUTS (beta coefficient = 0.096, p<0.001), prostate size and postvoid residual (PVR) but not with the storage symptoms of LUTS (beta coefficient = 0.015, p = 0.209) (Table 4).

**Table 4:** Multiple linear regression analysis of IPSS and associated factors affecting postmicturition dribble.

	Beta coefficient	p-value
Age	-0.009	0.81
Prostate size	0.007	0.015
PSA	-0.009	0.365
Qmax	0.013	0.103
PVR	-0.002	0.001
Voiding IPSS	0.096	0.000
Storage IPSS	0.015	0.209

IPSS, International Prostate Symptom Score; PSA, prostate specific antigen; PVR, postvoid residual

### 7. Association between the degree of PMD and IPSS-QoL score

After adjusting for age and the BPH-related factors, the degree of PMD was significantly correlated with the IPSS-QoL score (r = 0.260, p<0.001).

## Discussion

LUTS are nonspecific symptoms that may occur secondary to a wide variety of disorders. [12] LUTS are divided into three groups, storage, voiding and postmicturition symptoms, according to the ICS. Postmicturition symptoms are experienced immediately after micturition and include the feeling of incomplete emptying and PMD. PMD refers to the involuntary loss of urine immediately after an individual has finished passing urine. [1]

PMD has been known to be secondary to a small amount of residual urine in either the bulbar or prostatic urethra that is normally "milked back" into the bladder at the end of micturition by the normal reflex of the bulbocavernous muscle. [2,3] In addition, because the prevalence of PMD increases with age in men, BPH or other associated mechanisms, including urine entrapment in an obstructed prostatic urethra or disturbance of the normal function of the bulbocavernous muscle due to enlargement of the prostate, can also cause PMD. However, no study has reported the relationship between PMD and BPH-related parameters.

To date, many urological studies have focused on the storage and voiding symptoms of LUTS, but PMD has received relatively little attention. PMD may be considered less common and less bothersome than voiding and storage LUTS based on large, population-based studies. [7,13] In addition, PMD is not included in the IPSS, which is the most widely used tool for the evaluation of LUTS worldwide. Nevertheless, there are no questionnaire tools that have been developed to assess PMD. Therefore, we planned to develop and validate the HPMDQ to manage PMD patients with a more objective system.

To date, only a few studies have been conducted on PMD in LUTS, and these studies focused on the prevalence of PMD rather than on the clinical significance of PMD. [4–8] In the Tampere Ageing Male Urologic Study (TAMUS), which included more than 3000 men aged 50–70 years, the prevalence of PMD was 63%. Of the affected patients, 76% had mild symptoms and 24% had moderate to severe symptoms. [14] Ten years later, in a study involving more than 7000 men aged 30–80 years from the same cohort, the prevalence of PMD, which was 58.1%, increased with age and remained relatively constant after the age of 60. Minor bother from postmicturition was common, but major bother was rare. [4] In 1997, a Swedish study used postal questionnaires to investigate a research population of more than 10,000 subjects aged 45–90 years. Approximately 30% of all males, regardless of age, experienced PMD, and only a slight pattern of increasing frequency of PMD with increasing age was reported. [15] The internet-based EpiLUTS study reported an overall PMD prevalence of 46%.[16] However, none of the studies on this topic to date have reported a high prevalence of PMD. In the Epic study, which used telephone interviews, the overall prevalence of PMD was relatively low at 5.5%.[13] In the Boston Area Community Health (BACH) study, which involved 2301 men aged 30–79 years, the overall prevalence of postmicturition symptoms was 11.8%.[7] These differences in PMD prevalence can likely be explained by the use of various different tools to assess PMD, including the Danish Prostatic Symptom Score (DAN-PSS-1) and questionnaires developed by researchers, because the assessment of PMD is not included in the IPSS. Additionally, in some studies, the participants were classified as symptomatic if they reported having symptoms at least 'sometimes' [16], but other studies used 'fairly often' to classify patients as symptomatic. [7]

In our study, the prevalence of PMD was 51% and increased with age, but after the age of 70, the prevalence slightly decreased. Actually, the prevalence of PMD was lower in the 70s rather than 40s. We guess that after 60s, the prevalence of PMD does not seem to be significant more, and it seems to be a result of their thinking that PMD as a natural senile change rather than a great bothersome. These results are consistent with those obtained by the TAMUS study, in which the prevalence of PMD was 58.1% and increased with age while remaining relatively constant above the age of 70. [4] In the present study, we used the HPMDQ to assess PMD, and the questions used to assess PMD were similar to those in the DAN-PSS-1, which was used in the TAMUS study.

Similar to other LUTS, PMD has been known to have a significant impact on quality of life. In a population-based study in Finland involving 1709 men aged 18–79 years, PMD was reported as one of the most prevalent symptoms causing moderate or severe bother among men. [10] In the TAMUS study, Pöyhönen et al. [6] found that younger men aged 30–40 years experienced the most bother from PMD. Consistent with previous studies, in the present study, approximately 80% of the men with PMD reported at least minor bother. In younger men aged 40–49 years, the prevalence of patients who reported moderate or major bother was significantly higher than that in the older age groups. The cause of this is unclear, but it is thought that there will be a difference in susceptibility to symptoms due to the symptoms beginning at relatively young age.

Most studies to date on PMD have been limited to investigating its prevalence, and the clinical significance of PMD has rarely been reported. The BACH study reported that postmicturition symptoms were more closely related to voiding symptoms than to storage symptoms. [7] In the present study, the HPMDQ total score was significantly related to the voiding symptoms of LUTS, prostate size and PVR but not to the storage symptoms. This result is very similar to the results of BACH study, so this result seems to indicate that there is evidence that HPMDQ can reflect PMD well.

IPSS is the most widely used tool for the evaluation of LUTS, but PMD cannot be assessed using the IPSS because there are no questions concerning PMD on the IPSS. Therefore, most studies have used the DAN-PSS-1 or questionnaires developed by researchers to assess PMD. The DAN-PSS-1 questionnaire consists of 12 questions related to LUTS. [17,18] Using the DAN-PSS-1 questionnaire, the severity and associated bother of PMD could be assessed, but the frequency and related quality of life could not be evaluated.

Therefore, we developed the HPMDQ, which is a self-administered questionnaire, to assess PMD. The HPMDQ was designed to allow for the evaluation of various aspects of PMD, including frequency, severity, bother, quality of life and response to treatment. For validation of the questionnaire, we collected data on various parameters and analyzed their correlations with the HPMDQ score. The HPMDQ score was statistically significantly different between the two groups for all questionnaire scores and total score. So, the HPMDQ score seems to have clinical usefulness in assessing the symptoms of PMD. In addition, the HPMDQ total score showed a statistically significant positive correlation with the paper test as a quantitative test of PMD. These results suggest that the HPMDQ total score may be of value as a tool to quantify the symptoms of PMD. It is expected that it will play a big role in following the response to treatment when any treatment is performed similar to the role of IPSS in BPH treatment. It may be a useful tool for further research and clinical practice for PMD.

## Conclusions

The HPMDQ, which consists of 5 questions (frequency, severity, bother, quality of life and response to treatment for PMD), has been developed and validated. In the future, we expect to develop evidence-based evaluation through an objective scoring system for diagnosis and treatment of PMD using the HPMDQ. However, further studies are needed to determine the clinical usefulness of the HPMDQ.

## Supporting information

S1 File The Hallym PostMicturition Dribble Questionnaire (HPMDQ). (DOCX)
