# Supplementary material for: Development and validation of a symptom assessment tool for postmicturition dribble: A prospective, multicenter, observational study in Korea
Source: PLoS One. 2019 Oct 11;14(10):e0223734. doi: 10.1371/journal.pone.0223734 (PMC6788737; doi:10.1371/journal.pone.0223734)
Supplement: S1 File — (DOCX) [file pone.0223734.s001.docx]

**S1 File. The Hallym PostMicturition Dribble Questionnaire (HPMDQ).**

1. Over the last month, how often have you experienced dribbling after voiding when you feel you have finished urination?

0: not at all

1: 1 out of 3 times

2: 2 out of 3 times

3. almost always or always

1-(1). How much is dribbled urine after voiding?

1: immediately after voiding, a little

2: immediately after voiding, a lot

3: after wearing underwear, a little

4: after wearing underwear, a lot

2. Do you feel frustrated because of dribbling after voiding, when you feel you have finished urination?

0: not at all

1: slightly

2: moderately

3: a lot

3. If you were to spend the rest of your life with dribbling after voiding when you feel you have finished urination, how would you feel about that?

0: not dissatisfied

1: slightly dissatisfied

2: moderately dissatisfied

3: very dissatisfied

4. Compared to before treatment, have you experienced improvement in dribbling after voiding when you feel you have finished urination?

0: not at all

1: slightly

2: moderately

3: a lot

**Hallym Post-voided dribbling (PVD) Questionnaire (Korean version)**

| **1. 지난 한달 동안, 소변을 다 보았다고 느낀 후에,**  **소변이 흘러나온 경우가 있었습니까?** | | | | | | | | |
| --- | --- | --- | --- | --- | --- | --- | --- | --- |
|  | | 0: 없음 | 1: 3번 중 1번 | | 2: 3번 중 2번 | | | 3: 항상 |
| **1-(1). 흘러나온 소변양은 어느 정도입니까?** | | | | | | | | |
|  | **소변을 보고 난 직후** | | | 1. 작은 양 | | 2. 많은 양 | | |
|  | **속옷에 묻는 경우** | | | 1. 작은 양 | | 2. 많은 양 | | |
| **2. 소변을 다 보았다고 느낀 후에 소변이 흘러나오는 것 때문에,**  **불편을 느끼십니까?** | | | | | | | | |
|  | | 0: 불편하지 않다 | 1: 약간 불편하다 | | 2: 보통 정도로 불편하다 | | | 3: 많이 불편하다 |
| **3. 만약 소변을 다 보았다고 느낀 후에 소변이 흘러나오는 증상이 지속된다면,**  **어떻게 생각하십니까?** | | | | | | | | |
|  | | 0: 불편하지 않다 | 1: 약간 불편하다 | | 2: 보통 정도로 불편하다 | | | 3: 매우 불편하다 |
| **4. 치료전과 비교하여, 소변을 다 보았다고 느낀 후에**  **소변이 흘러나오는 증상의 호전이 있었습니까?** | | | | | | | | |
|  | | 0: 호전이 없다 | 1: 약간 호전되었다 | | 2: 보통 정도로 호전되었다 | | 3: 많이 호전되었다 | |
